# Temporal Regulation of Foregut Development by HTZ-1/H2A.Z and PHA-4/FoxA 

**DOI:** 10.1371/journal.pgen.0020161

**Published:** 2006-09-29

**Authors:** Dustin L Updike, Susan E Mango

**Affiliations:** Department of Oncological Sciences, Huntsman Cancer Institute, University of Utah, Salt Lake City, Utah, United States of America; The Jackson Laboratory, United States of America

## Abstract

The histone variant H2A.Z is evolutionarily conserved and plays an essential role in mice, *Drosophila,* and *Tetrahymena*. The essential function of H2A.Z is unknown, with some studies suggesting a role in transcriptional repression and others in activation. Here we show that Caenorhabditis elegans HTZ-1/H2A.Z and the remodeling complex MYS-1/ESA1–SSL-1/SWR1 synergize with the FoxA transcription factor PHA-4 to coordinate temporal gene expression during foregut development. We observe dramatic genetic interactions between *pha-4* and *htz-1, mys-1,* and *ssl-1*. A survey of transcription factors reveals that this interaction is specific, and thus *pha-4* is acutely sensitive to reductions in these three proteins. Using a nuclear spot assay to visualize HTZ-1 in living embryos as organogenesis proceeds, we show that HTZ-1 is recruited to foregut promoters at the time of transcriptional onset, and this recruitment requires PHA-4. Loss of *htz-1* by RNAi is lethal and leads to delayed expression of a subset of foregut genes. Thus, the effects of PHA-4 on temporal regulation can be explained in part by recruitment of HTZ-1 to target promoters. We suggest PHA-4 and HTZ-1 coordinate temporal gene expression by modulating the chromatin environment.

## Synopsis

During development, a single fertilized egg gives rise to the different cell types within an embryo. These different cell types are characterized by the different genes that they express. A critical question in biology is how embryonic cells activate genes at the appropriate time and place to generate the different cell types. In this paper, the authors explore temporal regulation of gene expression during formation of an organ, namely the Caenorhabditis elegans foregut. They have discovered that foregut genes require a variant of the canonical H2A histone for the onset of transcription. This variant, called H2A.Z, or htz-1 in *C. elegans,* promotes transcription by modifying how DNA is packaged within cells. Their data suggest that a key regulator of foregut development, the transcription factor PHA-4, recruits HTZ-1 to pharyngeal promoters, and this association contributes to their timely activation.

## Introduction

In eukaryotes, DNA and histones are assembled into nucleosomes, which present a daunting barrier to the transcriptional apparatus. The transcriptional machinery uses several approaches to augment or disrupt the repressive activity of chromatin, one of which is the exchange of canonical histones for histone variants. There are multiple variants of the core histone H2A, including MacroH2A, which is involved in mammalian X inactivation, H2A.X for DNA repair, and H2A.Z, whose function is linked to transcription [1]. Early studies with H2A.Z in *Tetrahymena* demonstrated that it was associated with the transcriptionally active macronucleus [2,3]. In yeast, H2A.Z has also been implicated in gene activation. H2A.Z antagonizes telomeric silencing and synergizes with the SWI/SNF remodeling complex to activate a subset of yeast genes [4]. In yeast, global chromatin binding studies have revealed that H2A.Z preferentially occupies promoter regions [5–8]. The function of this occupancy is currently being debated as some studies have found no correlation between H2A.Z occupancy and transcription rate [8], whereas others have suggested a strong inverse correlation between the presence of H2A.Z and the rate of transcription [5–7]. Despite these differences, the general consensus is that yeast H2A.Z configures chromatin to poise genes for transcriptional activation.

In metazoans, H2A.Z has been implicated in transcriptional repression more than activation. H2A.Z behaves genetically like a Polycomb group gene in *Drosophila* and is required for the stable association of Polycomb with polytene chromosomes [9]. In both mice and flies, H2A.Z localizes to heterochromatin, as well as euchromatin, and can associate with the heterochromatin protein HP1 [10,11]. One possibility is that H2A.Z enrichment in heterochromatin might prevent this domain from spreading. For example, H2A.Z is enriched in the 5′ insulator of the chicken β-globin locus that flanks a heterochromatic region [12]. However, this explanation is hard to resolve with a Polycomb-like function in *Drosophila*. These alternative roles may explain why vertebrate H2A.Z has diverged from yeast H2A.Z (61% similarity [7]) despite the general conservation of this histone variant in all eukaryotes [13]. Thus, the role of H2A.Z in metazoans remains unclear.

H2A.Z has not been studied previously in *Caenorhabditis elegans,* but the complex that assembles H2A.Z into chromatin in other organisms, the Esa1-Swr1 complex in yeast or the human SRCAP complex [13], was identified in C. elegans in a screen for regulators of vulval development [14]. Inactivation of Esa1–Swr1 homologs in C. elegans enhance mutations in known repressor complexes such as NuRD and Rb to de-repress the *lag-2/Delta* ligand during vulval development [14,15]. These observations raise the question of whether yeast and metazoans have evolved divergent functions for H2A.Z. One difficulty addressing this question is the paucity of direct H2A.Z target genes in metazoans, and the lack of knowledge of how and when H2A.Z is recruited to specific regions of DNA. Here we address these issues by examining the role of H2A.Z during C. elegans foregut (pharynx) development and the interplay of H2A.Z with the pharynx selector gene *pha-4*.

PHA-4 belongs to the FoxA family of transcription factors, which are critical to form the digestive tract during embryogenesis and also regulate metabolism during post-embryonic life [16,17]. In *C. elegans, pha-4* is required to specify cells of the pharynx during the earliest stages of organogenesis, and is also important later for organ differentiation, morphogenesis, and function [18,19]. This global role reflects the broad range of PHA-4 target genes. PHA-4 directly activates foregut genes expressed both early and late during foregut development and is required during post-embryonic life as well [18,20–22]. Thus, a key question is how diverse transcriptional responses are orchestrated by a single transcription factor. Previous studies have shown that the affinity of PHA-4 for its DNA binding site is one input for temporal regulation: promoters with high-affinity sites are competent to be expressed early whereas those with low-affinity sites are typically activated at later times [18,19]. A second strategy is combinatorial regulation of foregut target promoters. In *C. elegans,* association of PHA-4 with a target is rarely sufficient for expression, and additional factors contribute towards the precise temporal or cell type activation of pharyngeal genes [19,20]. Similarly, in vertebrates, expression in the liver critically depends on FoxA proteins that function in combination with additional liver transcription factors [17]. Here we explore a third input involved in the timely activation of pharyngeal genes: co-factors that are recruited to pharyngeal promoters.

FoxA proteins are thought to modify the chromatin environment of their target genes [17]. Gualdi et al. proposed that FoxA proteins function as competence factors that bind to promoters before they are transcriptionally active [23]. This association is thought to decompact nucleosomal DNA, which allows additional transcription factors to find their binding sites, leading to transcriptional activation [24]. The mechanism by which FoxA proteins modulate chromatin is still murky. Studies in vitro have largely focused on how FoxA itself can interact with nucleosomal DNA, and have not addressed the contribution of additional components. Here we explore the role of the histone variant HTZ-1/H2A.Z for pharyngeal gene activation, recruitment by PHA-4, and timing of expression.

## Results

### Requirement of *ssl-1, mys-1,* and *htz-1* for Pharyngeal Development

To find genes involved in pharynx development, we had previously created a temperature-sensitive configuration of *pha-4* that would allow us to examine suppressors and enhancers of compromised *pha-4* [25]. The *pha-4(ts)* strain contains the *pha-4(zu225)* allele, which carries a premature stop codon and renders *pha-4* mRNA subject to degradation by the nonsense-mediated decay pathway [25–27]. We took advantage of a temperature-sensitive allele of the nonsense-mediated decay pathway component, *smg-1(cc546ts)* [28], to modulate the accumulation of truncated PHA-4 by varying the temperature. At 15 °C, SMG-1 is active and mRNA derived from *pha-4(zu225)* is degraded, which results in Pha-4 lethality at the first larval stage (L1). At the permissive temperature of 24 °C, SMG-1 is compromised and *pha-4* mRNA is stabilized, which allows truncated PHA-4 to accumulate and worms to survive. At 20 °C, an intermediate level of truncated PHA-4 accumulates, producing a less severe pharyngeal phenotype that is nonetheless lethal. The *pha-4(ts)* strain provided a sensitive means to examine genetic interactions between *pha-4* and other loci, in particular the ability to test for enhanced lethality at permissive temperature or suppressed lethality at intermediate or restrictive temperatures.

**Figure 1:**
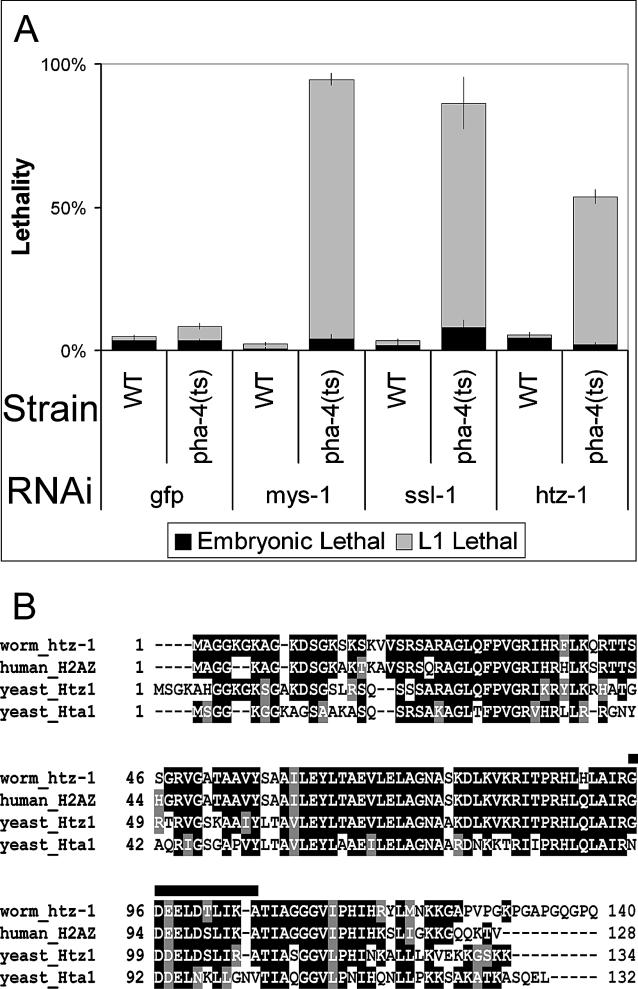
Enhancement of *pha-4(ts)* by *mys-1, ssl-1,* and *htz-1* (A) Feeding dsRNA to wild-type (WT) or *pha-4(ts)* [18,25] worms at the permissive temperature of 24 °C. WT worms generate viable progeny with *mys-1, ssl-1* or *htz-1* RNAi. In the *pha-4(ts)* background, L1 arrest increased with *mys-1, ssl-1,* or *htz-1* RNAi compared to control *GFP(RNAi)* (grey bars). Embryonic lethality remained unchanged (black bars). Effectiveness of RNAi feeding was manifest through viable, but sterile, progeny for *mys-1* and *ssl-1* [14], as well as repeated enhancement of L1 lethality for *pha-4(ts),* performed in parallel. *n* = 100 worms/plate, three plates per column. Error bars indicate the standard deviation. (B) Alignment of *C. elegans htz-1* (R08C7.3) with human H2A.Z, yeast Htz1, and one of the core H2A genes from yeast, Hta1. Extended acid patch region essential for H2A.Z function is indicated by the bar [50,60,67].

To investigate whether C. elegans homologs of the Esa1-Swr1 complex (known as *mys-1* and *ssl-1* [14]), were involved in pharynx development, we inactivated each of these genes using RNAi either alone or in combination with *pha-4(ts)*. Disrupting these genes by feeding worms bacteria expressing dsRNA [29] had no effect on viability (Figure 1A), and most animals developed into sterile adults, as had been observed previously [14,30,31]. Likewise, *pha-4(ts)* with control *GFP(RNAi)* was largely viable at permissive temperature (24 °C) (Figure 1A). However, when *pha-4(ts)* was combined with either *mys-1(RNAi)* or *ssl-1(RNAi)*, we observed a dramatic enhancement of lethality at L1 (Figure 1A). *htz-1(RNAi)* alone had no effect on viability [30], whereas we observed enhanced L1 lethality for *pha-4(ts)*; *htz-1(RNAi)* animals (Figure 1A and 1B, and Figure S1), consistent with the idea that SSL-1/Swr1 likely interacts with HTZ-1 in *C. elegans,* as it does in other organisms [32,33]. Importantly, *htz-1* has little sequence identity at the nucleotide level with H2A genes that could lead to “off-target” effects [34]. Moreover, we observed enhancement of *pha-4(ts)* lethality with double-stranded RNA (dsRNA) for the 3′UTR of *htz-1,* which lacks sequence identity with H2A genes (unpublished data). These data reveal that loss of *htz-1* is synthetically lethal with *pha-4(ts).*

**Figure 2:**
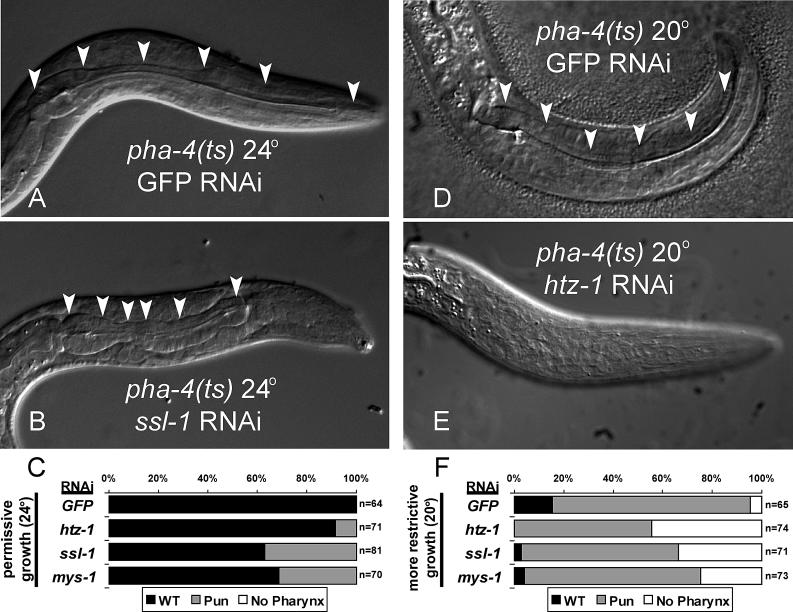
*mys-1, ssl-1,* and *htz-1* Enhance Pharyngeal Defects of *pha-4(ts)* (A–C) Feeding dsRNA to *pha-4(ts)* worms at the permissive temperature of 24 °C. (A) A *pha-4(ts); GFP(RNAi)* L1 with a wild-type pharynx (arrowheads). (B) A *pha-4(ts); ssl-1(RNAi)* L1 with an unattached pharynx (arrowheads). (C) Quantitation of *pha-4(ts)* animals exhibiting a normal pharynx (WT), an unattached or incomplete pharynx (Pun), or no detectable pharynx. *htz-1, ssl-1,* or *mys-1* RNAi significantly increased the number of Pun *pha-4(ts)* animals (*htz-1: p*=0.0290; *ssl-1* and *mys-1: p* < 0.0001, Fisher exact test). (D–F) Feeding dsRNA to *pha-4(ts)* animals at the intermediate temperature of 20 °C. (D) A *pha-4(ts); GFP(RNAi)* L1 at the intermediate temperature of 20 °C with a morphologically wild-type pharynx (arrowheads). (E) A *pha-4(ts); htz-1(RNAi)* worm at 20 °C missing a detectable pharynx. (F) Quantitation of *pha-4(ts)* animals exhibiting a normal pharynx (WT), an unattached or incomplete pharynx (Pun), or no detectable pharynx. *htz-1, ssl-1,* or *mys-1* RNAi significantly increased the number of worms with no detectable pharynx (*htz-1* and *ssl-1: p* < 0.0001; *mys-1: p* = 0.0015; Fisher exact test). WT worms with reduced *htz-1, ssl-1,* or *mys-1* activity had a wild-type pharynx at 24 °C and 20 °C (unpublished data). RNAi was conducted by feeding dsRNA [29].

*pha-4(ts); mys-1(RNAi), pha-4(ts); ssl-1(RNAi),* and *pha-4(ts); htz-1(RNAi)* larvae raised at both the permissive temperature (24 °C) and the intermediate temperature (20 °C) exhibited pronounced defects in pharynx development. At permissive temperature, RNAi against either *mys-1, ssl-1,* or *htz-1* generated Pun (pharynx unattached) *pha-4(ts)* larvae not observed with the *pha-4(ts); GFP(RNAi)* control (Figure 2A–2C). At intermediate temperature, RNAi against *mys-1, ssl-1, or htz-1* increased the percentage of L1 larvae lacking any detectable pharynx (Figure 2D–2F). For example, 44.6% of *pha-4(ts); htz-1(RNAi)* animals had no detectable pharynx (*n* = 74, Figure 2E and 2F), similar to *pha-4* null alleles [35]. By comparison, only 4.6% of *pha-4(ts); GFP(RNAi)* control larvae lacked an obvious pharynx (*n* = 65, Figure 2D and 2F). Other tissues appeared normal in *pha-4(ts); mys-1(RNAi), pha-4(ts); ssl-1(RNAi),* or *pha-4(ts); htz-1(RNAi)* larvae, as viewed under the light microscope. These data indicate that enhancement of *pha-4* lethality by *mys-1, ssl-1,* or *htz-1* reflects, at least in part, a defect in pharyngeal development.

**Figure 3:**
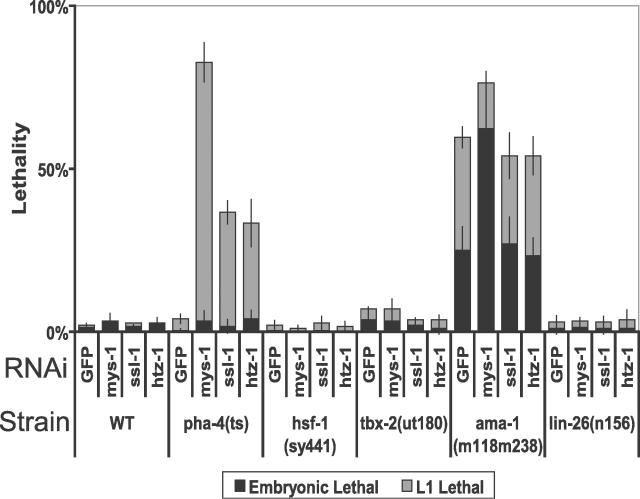
Specificity of *mys-1, ssl-1,* and *htz-1* Synergy with *pha-4(ts)* The indicated worm strains were fed dsRNA for GFP (negative control) or *mys-1, ssl-1,* or *htz-1* at 20 °C (or 24 °C for WT and *pha-4(ts)*)*.* Lethal embryos (black bars) or lethal L1 progeny (grey bars) were scored for each strain. Effectiveness of RNAi feeding was manifest through viable, but sterile, progeny for *mys-1* and *ssl-1* [14], as well as repeated enhancement of L1 lethality for *pha-4(ts). n* =1 00 worms/plate, three plates per column. Error bars indicate the standard deviation.

The synergy of *ssl-1, mys-1,* and *htz-1* with *pha-4* was specific since we did not observe enhancement by *ssl-1, mys-1,* or *htz-1* with strains carrying other compromised transcription factors (Figure 3). First we examined the heat-shock factor *hsf-1,* since H2A.Z binds some heat-shock promoters in *Drosophila* and yeast [5,10]. Normally, *hsf-1(sy441)* worms exhibit a temperature-sensitive developmental arrest at 25 °C and are viable at 15 °C [36]. We observed a low level of L1 lethality for *hsf-1(sy441); GFP(RNAi)* at the intermediate temperature of 20 °C. However, lethality was not enhanced by *ssl-1, mys-1,* or *htz-1* RNAi with *hsf-1(sy441)*. Null alleles of *tbx-2* and *lin-26* are each lethal [37,38], whereas *tbx-2(ut180)* or *lin-26(n156)* mutants survive with neuronal or vulval defects, respectively [39]. We did not detect increased lethality when *ssl-1, mys-1,* or *htz-1* was inactivated in combination with *tbx-2(ut180)* or *lin-26(n156)*. Unlike *pha-4(ts),* percent lethality for these mutants remained similar to the level observed with *GFP(RNAi).* Finally, we examined *ama-1,* which encodes the large subunit of C. elegans RNA polymerase II [40]. *ama-1(m118m238)* worms were sick either as single mutants or in combination with control *GFP(RNAi),* and segregated many embryonic and L1-arrested animals (Figure 3). Strikingly, neither *ssl-1* nor *htz-1* RNAi enhanced the lethality associated with *ama-1(m118m238)*. However, embryonic lethality was significantly increased with *mys-1(RNAi),* suggesting that *mys-1* may have additional roles that are independent of *ssl-1* and *htz-1*. Thus, strains exhibiting comparable baseline lethality to *pha-4(ts)* (i.e., *hsf-1, tbx-2,* and *lin-26*) or significantly higher baseline lethality *(ama-1)* did not exhibit enhanced lethality with either *htz-1* or *ssl-1*. We conclude that *pha-4* is exceptionally sensitive to reduction of *mys-1, ssl-1,* or *htz-1*.

As a second test for specificity, we examined a temperature-sensitive configuration of *unc-54,* which mimicked the *pha-4(ts)* strain. Like *pha-4(ts),* RNA derived from *unc-54(r293)* is subject to degradation by the nonsense-mediated decay pathway [27,28]. Combining *smg-1(cc546ts)* with *unc-54* leads to stabilization of *unc-54* mRNA at 24 °C and degradation at 15 °C [25,26]. Inactivation of *ssl-1, mys-1,* or *htz-1* with *unc-54(ts)* did not enhance the Unc-54 phenotype at permissive temperature or suppress *unc-54* at intermediate temperature (Figure S2). These results demonstrate that enhancement of *pha-4(ts)* by *ssl-1, mys-1,* and *htz-1* does not reflect indirect effects on the nonsense-mediated decay pathway. These data implicate roles for chromatin remodeling and histone exchange in the positive regulation of pharyngeal development.

### Association of HTZ-1/H2A.Z with Pharyngeal Promoters

We wondered what was the basis for the synthetic interaction between *htz-1* and *pha-4*. One intriguing possibility was that HTZ-1 might be recruited to pharyngeal promoters, which depend on PHA-4 for activation [18]. To address this idea, we used a nuclear spot assay [41,42] to visualize HTZ-1 association with target promoters in living embryos. We chose the nuclear spot assay because it allowed us to examine living embryos at precise times in development. We tagged the amino terminus of HTZ-1 with YFP and placed this chimera under control of the *htz-1* promoter. We also created a LacI::CFP construct [43] driven by the *htz-1* promoter. These two constructs were used to create transgenic worms that expressed both YFP::HTZ-1 and LacI::CFP from an extrachromosomal array. Because there were no mutations available, we could not assess whether our YFP::HTZ-1 reporter had rescuing activity. However, we believe the construct is functional since (1) we placed YFP at the equivalent location to a functional HA::Htz1 reporter in yeast [44], (2) the YFP::HTZ-1 localized with chromosomes in mitotic cells (Figure S3), and (3) YFP::HTZ-1 was distributed non-homogenously in interphase nuclei, similar to reports for endogenous H2A.Z in other organisms [10,11].

To observe association, we introduced a second extrachromosomal array (the reporter array) into the strain bearing YFP::HTZ-1 and LacI::CFP. The reporter array contained multiple copies of LacO operator sequences and a target promoter of interest. Three independent lines were generated in which the reporter array contained no target promoter. One line was generated with a reporter array containing the divergent heat-shock promoter for the *hsp-16.1* and *hsp-16.48* genes [45]. Three lines were generated in which the reporter array contained the promoter sequences for pharyngeally expressed *myo-2* [46], and two lines were created with a reporter array containing the pharyngeal promoter for *R07B1.9* [19]. Both *myo-2* and *R07B1.9* are PHA-4 target genes [18,19,21]. A single 0.7-μ confocal section was taken blindly through the center of an embryo (and pharynx) of an equal number of comma, 1.5-fold, 2-fold, and 3-fold stage embryos (see [47] for embryonic stages). This section typically bisected the nucleus of about 20 pharyngeal cells. Binding of LacI::CFP to LacO enabled us to localize the position of the extrachromosomal reporter array within the nucleus. Association of YFP::HTZ-1 with the reporter array was visualized as a bright dot co-localized with LacI::CFP. We scored the proportion of embryos with co-localized dots. To quantify our data, we counted the number of embryos with dots, rather than individual cells, because we could not always distinguish between individual cells when cells were packed closely together.

**Figure 4:**
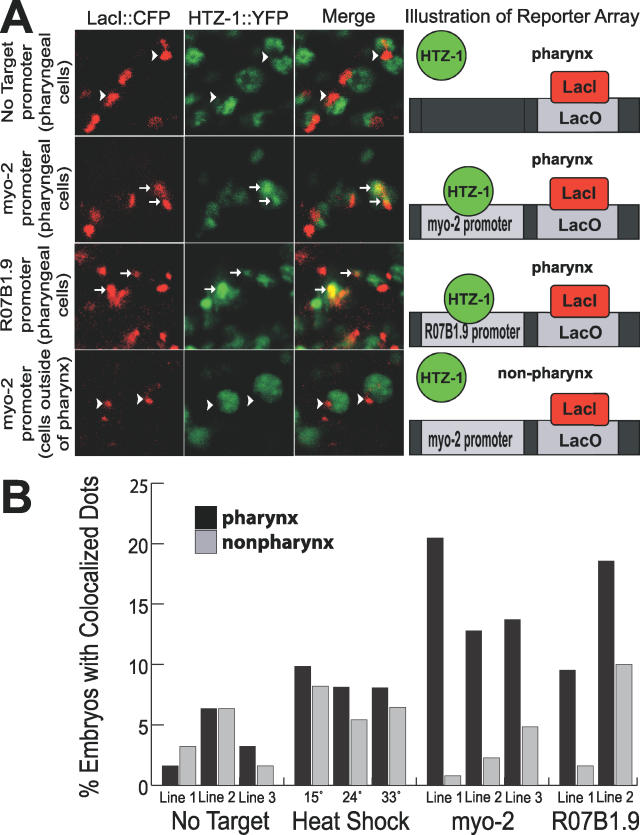
Association of YFP::HTZ-1 with Pharyngeal Promoters (A) Extrachromosomal target arrays were visualized by LacI::CFP (red) bound to the Lac operator (LacO). YFP::HTZ-1 (green) was excluded (arrowheads) from arrays with no-target promoter (row 1), but associated with target arrays containing promoters for pharyngeal genes *myo-2* or *R07B1.9* in pharyngeal cells *(*rows 2 and 3). Merge is yellow. YFP::HTZ-1 was excluded from arrays containing the *myo-2* promoter in non-pharyngeal cells (row 4). Cartoons depict interpretation of data. (B) Percentage of embryos containing one or more co-localized LacI::CFP and YFP::HTZ-1 dots in the pharynx (black) or outside of the pharynx (grey). Association was significantly higher in the three *myo-2* promoter lines (*p* < 0.0001) and two R07B1.9 lines (*p* = 0.0014). No significant difference in association was found when comparing the no-target lines to the heat-shock promoter target at 15 °C, 24 °C, or heat-shock at 33 °C for 30 min (*p* > 0.095). More than 60 embryos were scored for each no-target, heat-shock, and *R07B1.9* line. More than 120 embryos were scored for each *myo-2* line. YFP::HTZ-1 association was predominantly pharyngeal for *myo-2* (*p* < 0.0001) and *R07B1.9* (*p* = 0.0407) target arrays, but not control arrays (*p* = 1.00). An equivalent number of images were taken at the 1.5-, 2-, and 3-fold stages of embryogenesis for each line. The *p*-values were calculated using Fisher exact test.

We observed YFP::HTZ-1 associated with pharyngeal promoters in developing embryos. In our negative control, YFP::HTZ-1 rarely bound to reporter arrays when no promoter was present (Figure 4A, row 1, and 4B). However, YFP::HTZ-1 localized to reporter arrays carrying promoter sequences for either *myo-2* or *R07B1.9,* both of which are selectively expressed in the pharynx [18,46] (Figure 4A, rows 2 and 3, and 4B). This association was significantly higher in the three *myo-2* promoter lines (*p* < 0.0001) and two *R07B1.9* lines (*p* = 0.0014) compared to the no-target promoter controls. Strikingly, the no-target reporter arrays typically excluded YFP::HTZ-1 (Figure 4A, row 1), in contrast to the enrichment seen for reporter arrays with pharyngeal promoters. These data reveal that YFP::HTZ-1 associates with pharyngeal promoters in vivo.

H2A.Z has been implicated in both transcriptional activation and repression, depending on the organism and the assay. Therefore, we wanted to determine whether HTZ-1 associated with active or repressed promoters in C. elegans. First, we compared association with pharyngeal promoters within pharyngeal versus non-pharyngeal cells. We observed a dramatic enrichment of embryos with spots within pharyngeal cells, despite the larger number of non-pharyngeal nuclei present in each embryo. (We estimate a maximum of 5–10 pharyngeal muscle nuclei versus 50–100 non-pharyngeal nuclei per optical section, Figure 4A, row 4, and 4B). YFP::HTZ-1 association was significantly pharyngeal for *myo-2* (*p* < 0.0001) and *R07B1.9* (*p* = 0.0407) (Figure 4B). These data indicate that pharyngeal promoters preferentially associate with YFP::HTZ-1 within pharyngeal cells. Thus, we observed increased YFP::HTZ-1 association in cells that express *myo-2* and *R07B1.9,* and rare association in cells in which these genes are permanently silent. These data suggest HTZ-1/H2A.Z is not involved in constitutive repression of pharyngeal genes.

By comparison, the no-target reporter array lines had a low level of YFP::HTZ-1 spots, and these were evenly distributed in both pharyngeal and non-pharyngeal cells (Figure 4B; *p* = 1.0000, three lines). We also observed an even distribution of pharyngeal and non-pharyngeal spots when we examined a line containing the divergent heat-shock promoters in the reporter array [48]. There was no significant increase in spots for the heat-shock promoter array compared to the no-target control, and YFP::HTZ-1 association did not change at different temperatures (15 °C, 24 °C, or after heat shock at 33 °C for 30 min; *p* > 0.095). Thus, not all promoters are enriched for HTZ-1, and pharyngeal cells do not promote non-specific association of HTZ-1 with reporter arrays.

**Figure 5:**
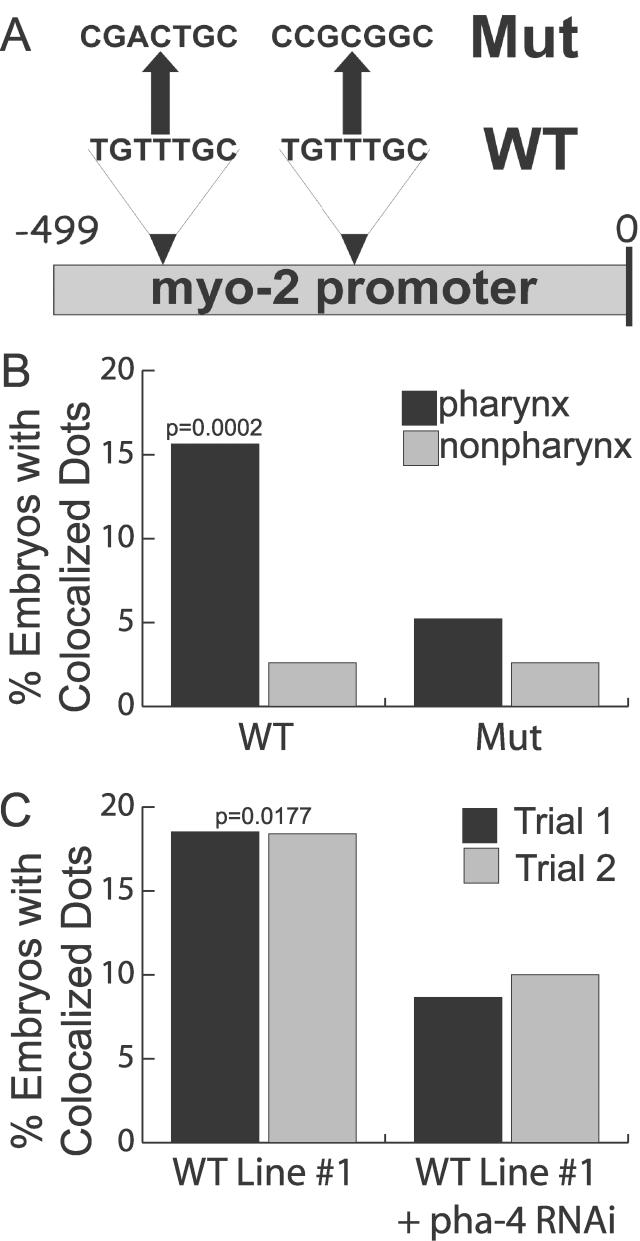
Association of YFP::HTZ-1 Depends on *pha-4* Activity (A) Two PHA-4 binding sites within the *myo-2* promoter were mutated in the *myo-2* Mut construct [18]. (B) YFP::HTZ-1 association in the pharynx (black) and outside of the pharynx (grey). *n* = 384 embryos for three wild-type (WT) lines and 192 embryos for three mutant (Mut) lines. *p*-value indicates a significant difference for pharyngeal association of YFP::HTZ-1 in WT versus Mut. (C) Association of YFP::HTZ-1 with the wild-type *myo-2* promoter (line #1) decreased in *pha-4(RNAi)* embryos within pharyngeal cells. An equivalent number of images were taken at the 1.5-, 2-, and 3-fold stages of embryogenesis and >80 embryos were imaged for each trial. *p*-values calculated using Fisher's exact test.

Our second test to explore roles in activation versus repression was to examine whether PHA-4 was required for YFP::HTZ-1 recruitment to pharyngeal promoters. We created three independent lines with reporter arrays containing a *myo-2* promoter with mutated PHA-4 binding sites (Figure 5A) [18]. Each of these lines showed a significant decrease in the percent of embryos with YFP::HTZ-1 occupancy on the reporter array in the pharynx (Figure 5B). This level resembled the degree of association observed in non-pharyngeal cells or lines bearing no-target reporter arrays (Figure 4B versus Figure 5B). Similarly, inactivation of *pha-4* by RNAi decreased the occupancy of YFP::HTZ-1 on *myo-2* sequences by one half (Figure 5C). These results revealed a requirement for *pha-4* for robust association of YFP::HTZ-1 with the *myo-2* promoter. The dependency on *pha-4* for YFP::HTZ-1 association supports the genetic synergy between *pha-4* and *htz-1* to promote pharyngeal development.

**Figure 6:**
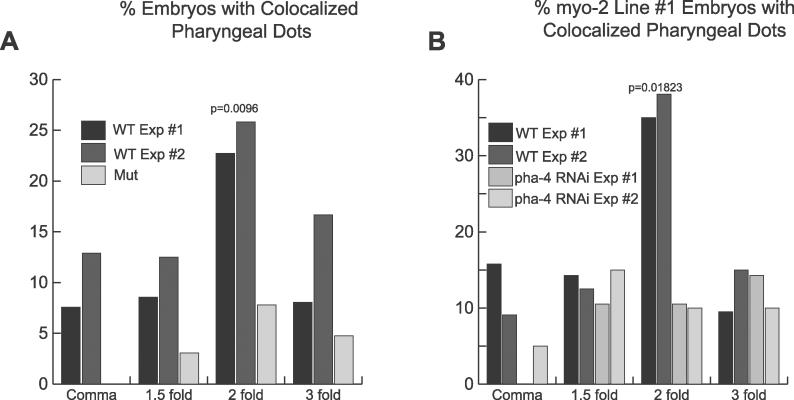
YFP::HTZ-1 Association Peaks at the Onset of *myo-2* Expression (A) Association of YFP::HTZ-1 with the *myo-2* promoter in the pharynx during the comma, 1.5-, 2-, and 3-fold stages of development (two trials: black, dark grey). YFP::HTZ-1 association in the pharynx does not peak at the 2-fold stage for a *myo-2* promoter with both PHA-4 binding sites mutated (Mut, light grey). *n* > 60 embryos imaged at each stage, for each experiment. Multiple lines were used for each trial. (B) Pharyngeal YFP::HTZ-1 association with *myo-2* target array #1 decreased at the 2-fold stage when *pha-4* activity was reduced by RNAi*. n* > 20 embryos imaged at each stage for each of the two wild-type (WT) and two *pha-4(RNAi)* experiments. The *p*-values for (A) and (B) indicate the significance of the WT peak at the 2-fold stage as calculated by a repeated measures analysis of variance (ANOVA).

Our third test to examine roles in activation versus repression was to determine when YFP::HTZ-1 associated with pharyngeal promoters. We examined embryos from the three *myo-2* target lines spanning 4 h of embryogenesis (comma, 1.5-, 2-, or 3-fold stages). At the 2-fold stage, the association of YFP::HTZ-1 nearly tripled above what was observed at the comma and 1.5-fold stages (Figure 6A and 6B). This peak required *pha-4* activity since it was lost when *pha-4* was inactivated by RNAi (Figure 6B) or when PHA-4 binding sites were mutated within the *myo-2* promoter (Figure 6A). YFP::HTZ-1 association with *myo-2* decreased to background levels after the gene was fully active at the 3-fold stage. Thus, YFP::HTZ-1 was enriched at the *myo-2* promoter at the developmental stage when *myo-2* transcription initiates [49].

### HTZ-1 Influences the Onset of Pharyngeal Gene Expression

**Figure 7:**
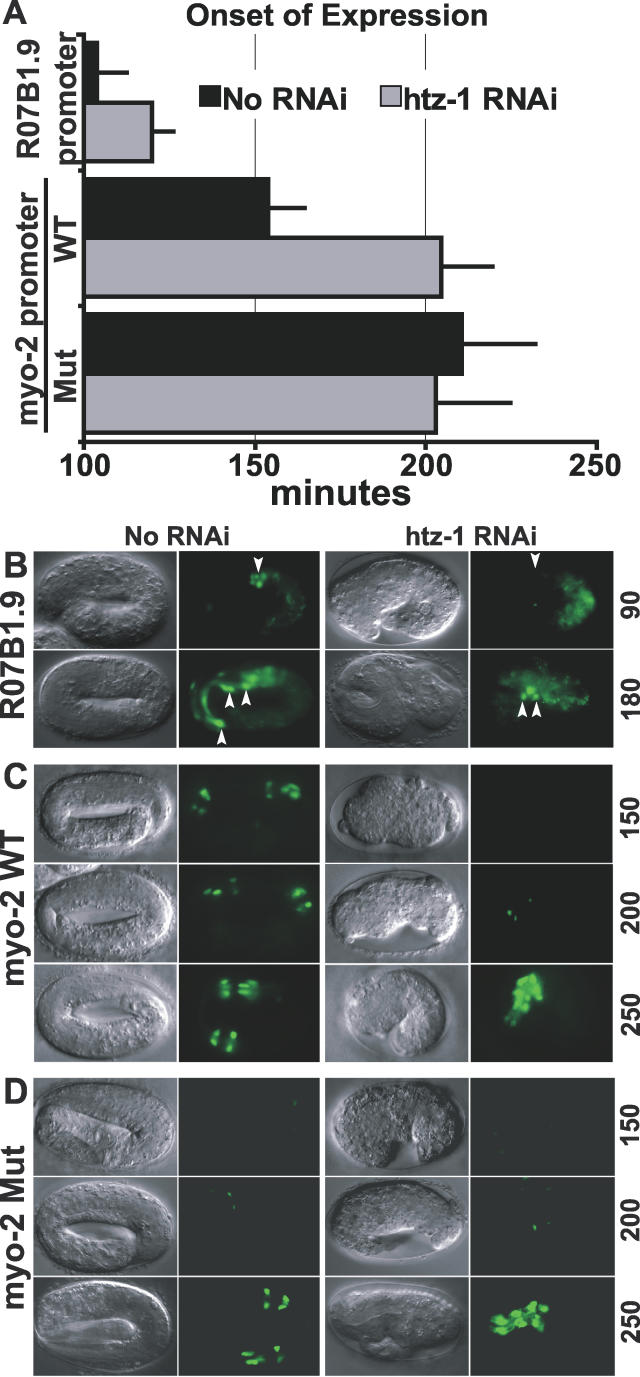
*htz-1* Influences the Onset of Late Pharyngeal Gene Expression (A) Onset of *R07B1.9::GFP* and *myo-2::*GFP after the comma stage for wild-type (black) or *htz-1(RNAi)* (grey) embryos. Activation of the *R07B1.9* reporter was delayed after injection of *htz-1* dsRNA (*p* < 0.0001, Student *t*-test). No RNAi: *n* = 18, *htz-1* RNAi: *n* = 12 embryos. *myo-2* activation was scored in embryos carrying either a wild-type *myo-2*::GFP reporter (WT) or a *myo-2::*GFP reporter with both PHA-4 binding sites mutated (Mut) [18]. Activation of the WT reporter was delayed after injection of *htz-1* dsRNA (*p* < 0.0001, Student *t*-test), whereas onset of the Mut reporter was unchanged (*p* = 0.2068). No RNAi: *n* = 15 WT, *n* = 33 Mut Embryos; *htz-1* RNAi: *n* = 9 WT, *n* = 21 Mut embryos. Error bars indicate the standard deviation. (B) Expression of *R07B1.9::GFP* at 90 and 180 min after the comma stage with *htz-1* RNAi or without (No RNAi). Pharyngeal *R07B1.9::GFP* is indicated by arrowheads. (C) Expression of *myo-2*::GFP (WT) at 150, 200, and 250 min after the comma stage with *htz-1* RNAi or without (No RNAi). (D) Expression of *myo-2*::GFP lacking PHA-4 binding sites [18] at 150, 200, 250 min after the comma stage with *htz-1* RNAi or without (No RNAi).

PHA-4 influences the timing of expression of genes within the pharynx, [18,19], raising the question of whether *htz-1* might also contribute to temporal regulation. We removed *htz-1* mRNA by microinjection of *htz-1* dsRNA and examined the onset of *R07B1.9*::GFP and *myo-2*::GFP expression (Figure 7A–7C). Microinjection was used over RNAi feeding since microinjection typically gives the strongest RNAi phenotype. For example, microinjection of dsRNA for *ssl-1* causes embryonic arrest, whereas feeding worms bacteria expressing dsRNA generates viable, sterile animals(Figure S4) [14].

Microinjection of *htz-1(RNAi)* caused highly penetrant, late-embryonic arrest (Figure 7B–7D), which phenocopied embryonic arrest from *ssl-1(RNAi)* microinjection (Figure S4). We focused on events after the comma stage of embryogenesis because *htz-1(RNAi)* embryos appeared to develop identically to the wild type up to the comma stage, based on morphological criteria (Figure S5)**.** After the 2-fold stage, embryos failed to elongate (Figures 7B–7D). In terminal embryos, the pharynx and intestine appeared differentiated, but misshapen morphologically (Figure S4).

*R07B1.9*::GFP was first activated 104 min after the comma stage in the wild type, whereas activation was delayed to 120 min in *htz-1(RNAi)* embryos (Figure 7A and 7B). Likewise, *myo-2*::GFP activation was delayed from 154 min after the comma stage in the wild type to 204 min in *htz-1(RNAi)* embryos (Figure 7A and 7C). The delay of *myo-2*::GFP expression with *htz-1(RNAi)* was similar to that seen when PHA-4 binding sites were mutated within the *myo-2* promoter (Figure 7A and 7D) [18]. *htz-1* was not essential to repress *R07B1.9* or *myo-2* expression at early stages of pharyngeal development (Figure 7B, 90 min, and 7C, 150 min), nor was it important to achieve the ultimate strong expression during the terminal stages of development (Figure 7B, 180 min, and 7C, 250 min). These data suggest that *htz-1* is critical for the timely activation of *R07B1.9* and *myo-2.* We note that these findings do not rule out a role for H2A.Z in transcriptional repression or synergy with other transcription factors in other contexts.

We observed no difference in GFP expression between wild-type and *htz-1(RNAi)* embryos for a *myo-2::*GFP reporter bearing mutated PHA-4 binding sites (Figure 7A and 7D, 200 min). This result indicates that the delay in *myo-2* activation after *htz-1(RNAi)* does not reflect non-specific effects of *htz-1(RNAi)* on embryonic development. In summary, we can account for the effects of PHA-4 on timing of *myo-2* activation [18] by its ability to recruit HTZ-1.

**Figure 8:**
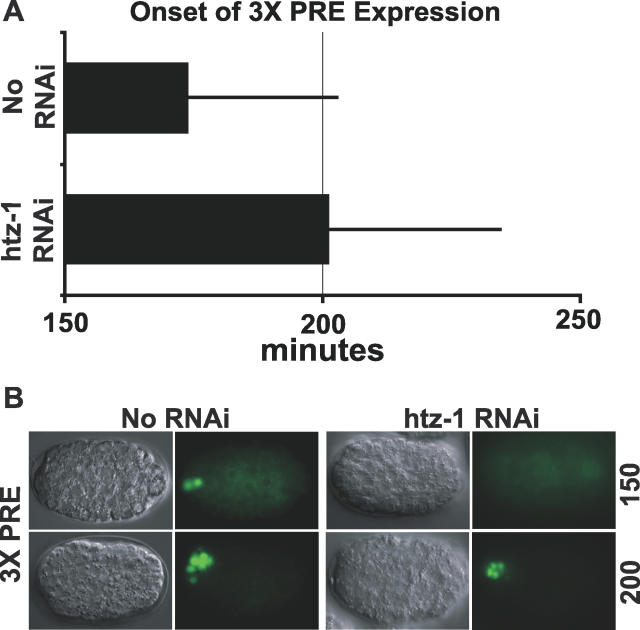
*htz-1* Influences the Onset of Early PHA-4–Dependent Expression (A) Onset of *3XPRE::GFP* after the two-cell stage for wild-type (black) or *htz-1(RNAi)* (grey) embryos*. 3XPRE::GFP* is a reporter construct with three copies of a high-affinity PHA-4 response element upstream of the *Δpes-10* promoter that reproducibly activates early pharyngeal expression [19]. Activation of the *3XPRE::GFP* reporter is delayed after injection of *htz-1* dsRNA (*p* = 0.0289, Student *t*-test). No RNAi: *n* = 23, *htz-1* RNAi: *n* = 9 embryos. Error bars indicate the standard deviation. (B) Expression of *3XPRE::GFP* at 150 and 200 min after the two-cell stage with *htz-1* RNAi or without (No RNAi).

We also examined the effects of *htz-1* on a GFP reporter driven by three copies of a high-affinity PHA-4 binding site (TGTTTGC) upstream of a minimal Δ*pes-10* promoter (3xPRE::GFP) [19]. This reporter reproducibly activates pharyngeal expression early, at the beginning of pharynx primordium formation [19]. Activation of 3xPRE::GFP was delayed from 174 min past the two-cell stage of embryogenesis in wild-type embryos to 201 min in *htz-1(RNAi)* embryos (Figure 8A and 8B). These data reveal that *htz-1* is important for the timely activation of genes within the pharynx at early as well as late stages of development.

## Discussion

Developmental events such as organogenesis are sensitive to precise temporal regulation, to integrate the processes of cell-fate specification, differentiation, and morphogenesis. Our studies with C. elegans have revealed three inputs that control temporal gene expression during pharyngeal organogenesis. The first is PHA-4 and its affinity for target promoters. PHA-4 functions globally to activate both early- and late-expressed pharyngeal genes. The affinity of PHA-4 for its DNA binding site contributes to the timing of expression of these targets. High-affinity binding sites promote expression during early organogenesis, whereas low-affinity sites are typically restricted to later development [18,19]. The second input for temporal control are *cis*-regulatory elements that function combinatorially with PHA-4. Enhancer and repressor elements have been discovered that promote either early or late pharyngeal gene expression, in combination with PHA-4 [19]. The third input, described here, is the histone variant HTZ-1. The data we present reveal that PHA-4 is required to recruit HTZ-1 to a subset of pharyngeal promoters, and that HTZ-1 is required for the timely onset of transcription. Thus, activation of pharyngeal genes by PHA-4 may depend on its ability to recruit HTZ-1.

### Roles for MYS-1, SSL-1, and HTZ-1

**Figure 9:**
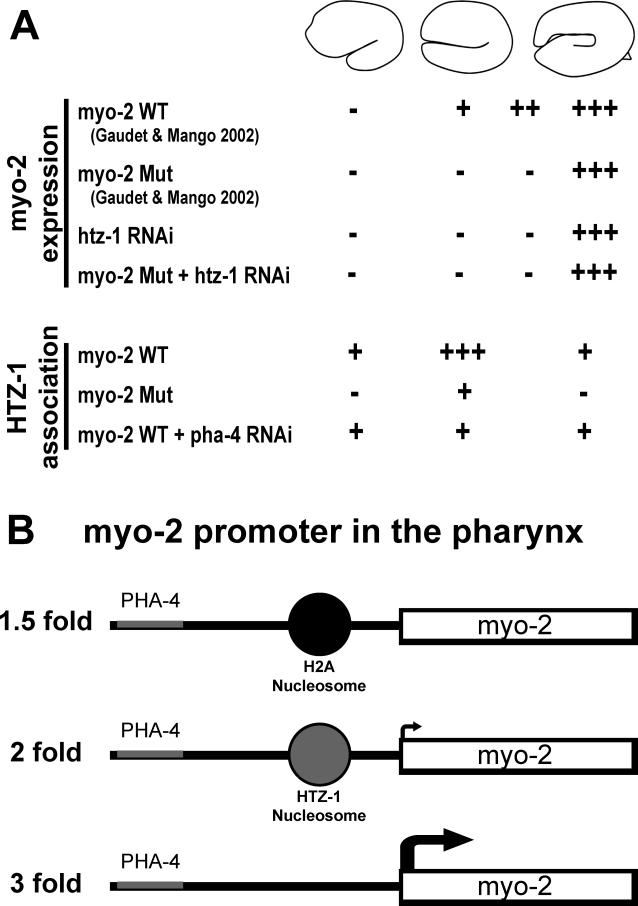
Summary and Model: HTZ-1 Synergizes with PHA-4 to Establish the Foregut (A) Summary of the data presented in this paper and in [18]. *myo-2* expression initiates at the 2-fold stage, and onset at this stage requires *pha-4* and *htz-1* because mutation of PHA-4 binding sites (Mut) or *htz-1* RNAi lead to a delay in *myo-2* activation. HTZ-1 association with the *myo-2* promoter peaks at the onset of *myo-2* transcription, and this association requires *pha-4*. (B) Model to explain how HTZ-1 synergizes with PHA-4. PHA-4 association with the *myo-2* promoter leads to exchange of H2A-containing nucleosomes for one or more nucleosomes carrying HTZ-1/H2A.Z at the 2-fold stage. Based on data from other organisms [1], we propose MYS-1 and/or SSL-1 function in a complex that performs the exchange reaction. HTZ-1–containing nucleosomes promote transcriptional activation by the 2-fold stage.

Our studies have revealed that *pha-4* synergizes with *mys-1/TIP60–ssl-1/swr1* and *htz-1/H2A.Z* to promote activation of pharyngeal gene expression during organogenesis. Partial inactivation of *pha-4* in combination with *htz-1, mys-1,* or *ssl-1* RNAi resulted in a specific, synergistic lethality. YFP::HTZ-1 associated with pharyngeal gene promoters. This association was particularly striking at the onset of transcription and required PHA-4. *htz-1* was required for the late onset of expression for the pharyngeal genes R07B1.9 and *myo-2,* and for the early onset of expression of 3xPRE. For *myo-2,* the association and transcriptional effects of HTZ-1 depended on PHA-4 binding sites and *pha-4* activity. Together, these data suggest that HTZ-1 promotes the timely activation of pharyngeal genes in response to PHA-4 (Figure 9).

How broad is the influence of HTZ-1 for transcriptional activation? We note that loss of *htz-1* function did not enhance *unc-54, hsf-1, tbx-2, ama-1,* or *lin-26*. Thus, some transcription appears relatively insensitive to loss of *htz-1.* Intriguingly, a genome-wide survey of Htz1 occupancy in yeast revealed a correlation between Htz1 and four transcription factors, one of which was the Fox homolog Fkh1 [5]. In *Xenopus,* endogenous H2A.Z is highly expressed in the notochord [50], a tissue that depends on the *pha-4* orthologue FoxA2 for establishment [51–53]. These observations suggest that certain transcription factors, including forkhead proteins, may be acutely dependent on H2A.Z.

The occupancy of HTZ-1 at pharyngeal promoters was particularly striking at the onset of transcription, but we also observed a small percentage of embryos with HTZ-1 association at other stages. One possibility is that there are two pools of HTZ-1: one that is constitutively associated with genes for an unknown function, and a second pool that is recruited transiently to promoters to facilitate their activation. The idea of distinct pools of H2A.Z is supported by studies in yeast that have examined H2A.Z location, acetylation, and Swr1 dependency [6,54–57]. Alternatively, it may be that the low, constitutive association reflects non-specific association with DNA or ambiguities with the nuclear spot assay. Future studies will be needed to tease these possibilities apart.

### FoxA and Chromatin Remodeling

FoxA transcription factors have been implicated in chromatin regulation. In vertebrates, FoxA2 associates with genes long before they are transcriptionally active and is thought to promote their transcriptional potentiation [17]. FoxA can decompact reconstituted H1-containing nucleosomal arrays in vitro [24], suggesting a mechanism by which FoxA could promote transcriptional activation in vivo. Our data suggest that H2A.Z contributes to FoxA modulation of chromatin. Previous studies implicated H2A.Z in decompaction of oligonucleosome arrays [58,59], and the crystal structure of H2A.Z revealed that a key interaction between H2A and H3 was disrupted in H2A.Z [60]. Thus, an interesting possibility is that H2A.Z contributes to decompaction of target promoters by FoxA proteins, perhaps by affecting the structure or stability of nucleosomes.

How many pharyngeal genes depend on HTZ-1? We examined the promoters of two pharyngeal genes, *myo-2* and *R07B1.9,* in detail. Although HTZ-1 associated with the promoters of these two target genes late in development, the lack of a detectable pharynx in many *pha-4(ts); htz-1(RNAi)* larvae cannot be attributed to *myo-2* and *R07B1.9*. Most likely, HTZ-1 works with PHA-4 at multiple targets and has synergistic effects on early as well as late pharyngeal genes (see, for example, 3xPRE). However, some pharyngeal genes may be independent of *htz-1.* For example, *htz-1(RNAi)* did not affect the timing of activation of *pax-1* (Figure S6), a gene that is expressed in pharyngeal marginal and epithelial cells (J. Stevenson, A. Chisholm, and S. E. Mango, unpublished data). Nor did we observe YFP::HTZ-1 associated with *pax-1* (*n* = 60 embryos; Figure S6). It will be intriguing to determine the characteristics of individual pharyngeal promoters that determine whether it is *htz-1* dependent or not.

YFP::HTZ-1 association with reporter arrays containing the *myo-2* promoter decreased at the 3-fold stage, when *myo-2* expression is at its peak [49]. One speculative explanation of this result is that HTZ-1 is lost from the *myo-2* promoter as transcription gets under way. In yeast, Htz1 is displaced from the Gal1 and Pho5 promoters after they become active [1]. Moreover, Htz1-containing nucleosomes are less stable than canonical, H2A-containing nucleosomes in vitro [5]. Thus, it is possible that prior to PHA-4 association, HTZ-1 is absent from *myo-2* because this promoter is not primed for activation, but at the 3-fold stage, when the gene is transcribed, HTZ-1 is absent because nucleosomes containing HTZ-1 have been evicted. On the other hand, in non-pharyngeal cells, no PHA-4 is available to bind *myo-2* or *R07B1.9,* and these genes do not recruit HTZ-1. This model suggests that pharyngeal genes pass through distinct states as they transition towards active transcription.

## Materials and Methods

### Strains and alleles.

C. elegans strains were maintained as described [61]. Strains include: SM190 *smg-1(cc546ts)I; pha-4(zu225)V* [18,25]; SM279 is PD8120 *smg-1(cc546ts)I* 5X out-crossed [25], PR1182 *cha-1(p1182)IV* [62], SM1353 *cha-1(p1182)IV; pxEx214(HTZ-1promoter::YFP::HTZ-1 + HTZ-1promoter::CFP::LacI +pRF4)*, SM1358 *cha-1(p1182)IV; pxEx214 pxEx219(cha-1 + LacO)*, SM1469 *cha-1(p1182)IV; pxEx214 pxEx240(cha-1 + LacO),* SM1470 *cha-1(p1182)IV; pxEx214 pxEx241(cha-1 + LacO),* SM1357 *cha-1(p1182)IV; pxEx214; pxEx218(cha-1 + HS prom + LacO),* SM1356 *cha-1(p1182)IV; pxEx214; pxEx217(myo-2 prom + LacO + cha-1),* SM1393 *cha-1(p1182)IV; pxEx214; pxEx222(myo-2 prom + LacO + cha-1),* SM1394 *cha-1(p1182)IV; pxEx214; pxEx223(myo-2 prom + LacO + cha-1),* SM1390 *cha-1(p1182)IV; pxEx214; pxEx220(myo-2 mut prom + LacO + cha-1),* SM1395 *cha-1(p1182)IV; pxEx214; pxEx224(myo-2 mut prom + LacO + cha-1),* SM1397 *cha-1(p1182)IV; pxEx214; pxEx233(myo-2 mut prom + LacO + cha-1),* SM1391 *cha-1(p1182)IV; pxEx214; pxEx221(R07B1.9 prom + LacO + cha-1),* SM1408 *cha-1(p1182)IV; pxEx214; pxEx227(R07B1.9 prom + LacO + cha-1),* SM1355 *cha-1(p1182)IV; pxEx214; pxEx216(pax-1 prom + LacO + cha-1),* SM1031 contains pxEx171(myo-2::GFP::His2B + pRF4), and SM1033 contains pxEx173(myo-2mut::GFP::His2B + pRF4) [18], SM202 pxIs2(pax-1::GFP +pRF4), SM1064 pxEx187(R07B1.9::GFP::His2B + pRF4) [20], SM1535 pxIs17(3XPRE::GFP::His2B + pRF4) [19], *unc-54(ts)* is PD8118 *smg-1(cc546ts) unc-54(r293)I,* PS3551 is *hsf-1(sy441)I,* JC1970 is *tbx-2(ut180)III,* DR685 was used to obtain *dpy-13(e184) ama-1(m118m236)IV,* and MT156 is *lin-26(n156)II*.

### Injections.

SM1353 *cha-1(p1182)IV; pxEx214(HTZ-1promoter::YFP::HTZ-1 + HTZ-1promoter::CFP::LacI +pRF4)* was created by injecting PR1182 with pRF4 (50 ng/μl) [63] linearized with EcoR1, HTZ-1P::YFP::HTZ-1 PCR product (0.5 ng/μl), HTZ-1P::CFP::LacI PCR product (4 ng/μl), and sheared herring sperm DNA to 100 ng/μl [64]. Target strains were created by injecting SM1353 with the KpnI/SphI lacO fragment from pSV2-dhfr-8.32 [43], 2 ng/μl of *cha-1* plasmid RM#527p linearized with ApaI, 10 ng/μl of linear target promoter, and sheared herring sperm DNA to 100 ng/μl. For *myo-2,* we used 511 base pairs of the endogenous promoter upstream of the start codon, the same length used in the *myo-2p::GFPHis2B* reporter. For *R07B1.9,* we used 1,000 base pairs of the endogenous promoter upstream of the start codon, the same length used in the *R07B1.9p::GFPHis2B* reporter. A total of 265 base pairs of the pax-1 endogenous promoter were used, the same length used in the pax-1::GFP reporter.

### Identification of enhancers of the partial loss of *pha-4* activity.

HT115 bacteria expressing dsRNA for GFP, *mys-1, ssl-1,* or *htz-1* from [30] were used for feeding RNAi according to standard procedures [29]. RNAi was initiated at the L4 stage and progeny scored 2–3 d later.

### Nuclear spot assay.

Images were acquired from live embryos using a LSM510 Meta confocal microscope. A multi-track setting was used to acquire CFP and YFP from a 0.7-μ section taken blindly from the center of the embryo (and pharynx), as determined by differential interference contrast (DIC) microscopy. Positives must have both YFP::HTZ-1 and subnuclear CFP::LacI foci. YFP::HTZ-1 foci are unevenly distributed throughout each nucleus (Figure S3). For YFP::HTZ-1 dots to be considered co-localized, CFP and YFP foci had to overlap and have the same shape. Dots that overlapped, but appeared to have distinct morphologies or locations within the nucleus, were not considered co-localized. Co-localized spots were counted in each embryo and classified as pharyngeal or non-pharyngeal based on position within the embryo as determined by light DIC image.

### Expression timing assay.

*htz-1* dsRNA [30] was injected into *myo-2*::GFP:*:His2B, myo-2Mut::*GF*P::His2B,* or *R07B1.9:*:GFP*::His2B* transgenic worms at a concentration of 3.2 μg/μl. Comma-stage embryos were picked and examined at 10-min intervals, and initial expression of *myo-2*::GFP::His2B, *myo-2Mut*::GFP::His2B, or R07B1.9::GFP::His2B scored at 22 °C. Only embryos with embryonic defects from *htz-1* RNAi were compared to uninjected hermaphrodites. *htz-1* dsRNA was injected into *3XPRE::GFP::His*2B and *pax-1::GFP* transgenic worms at a concentration of 3.2 μg/μl. Injected worms were dissected the next day, and two-cell embryos were picked and examined at 10-min intervals at 22 °C.

### Immunostaining.

Embryos were stained as described previously [65,66]. Antibodies used were polyclonal mouse-generated αHTZ-1 (Bill Kelly [Emory]; B. Kelly, unpublished data) at a dilution of 1:500 and rabbit anti-TBP [65] at a dilution of 1:20.

### Crawl assay.

Bacterial plates for GFP, *mys-1, ssl-1,* or *htz-1* RNAi were made as described in the body of the text. Five worms of the genotype *smg-1(cc546ts) unc-54(r293),* called *unc-54(ts),* were picked to two of each GFP, *mys-1, ssl-1,* or *htz-1* RNAi plates and incubated at 20 °C or 24 °C. Three days later, four F1 progeny from each plate were picked to a new plate, allowed to crawl for 10 min, and then photographed. As a control, *smg-1(cc546ts); pha-4(zu225)*, called *pha-4(ts),* animals were put under the same conditions at the same time. L1 lethality was replicated, as in Figure 1A and Figure 2, at 96% with *mys-1,* 92% with *ssl-1,* and 48% with *htz-1* RNAi (*n* = 25/strain).

## Supporting Information

Figure S1 *htz-1(RNAi)* by Bacterial Feeding Reduces HTZ-1 Levels Cells co-stained with rabbit anti-TBP [65] and DAPI (*n* = 5). HTZ-1 antibody was a generous donation from Bill Kelly, Emory University; B. Kelly, unpublished data.
(2.4 MB PDF)

Figure S2 *mys-1, ssl-1,* and *htz-1* Do Not Enhance *unc-54(ts)*
*unc-54* activity was monitored by a crawling assay at the intermediate temperature of 20 °C or the permissive temperature of 24 °C. After RNAi against the indicated gene, four *unc-54(ts)* worms were picked to a new plate and allowed to crawl for 10 min. At 24 °C, *unc-54(ts)* worms were mobile, and this movement was not inhibited when *mys-1, ssl-1,* or *htz-1* were inactivated. At 20 °C, *unc-54(ts)* worms were largely immobile, and movement was not improved by *mys-1, ssl-1,* or *htz-1* RNAi.
(4.7 MB PDF)

Figure S3 YFP::HTZ-1 Expression in a C. elegans Embryo YFP::HTZ-1 associates with chromatin in cells during metaphase and anaphase (arrows). YFP::HTZ-1 is non-randomly distributed in cells during interphase (asterisk), similar to other organisms [10].
(2.9 MB PDF)

Figure S4 Late-Embryonic Lethality of *htz-1(RNAi)* Phenocopies Lethality of *ssl-1(RNAi)*
(A) Microinjection of 3.2 μg/μl of *htz-1* dsRNA caused a highly penetrant late-embryonic arrest. Four terminal *htz-1(RNAi)* embryos show the pharynx (dotted red line) and intestine appear differentiated, but misshapen morphologically.
(B) Microinjection of 0.5 μg/μl of *ssl-1* dsRNA caused a highly penetrant late-embryonic arrest that is indistinguishable from *htz-1(RNAi)* under the light microscope. Four terminal *ssl-1(RNAi)* embryos show that the pharynx (dotted-red line) and intestine appear differentiated, but misshapen morphologically. Embryos from injected worms were picked to a new plate after 1 d and allowed to develop 18 h before imaging.
(8.3 MB PDF)

Figure S5 *htz-1(RNAi)* Has No Effect on Timing of Early Embryonic Development Two-cell embryos were dissected from uninjected hermaphrodites and hermaphrodites injected 2 d prior with 3.2 μg/μl of double-stranded *htz-1* RNA. Only embryos with embryonic defects from *htz-1(RNAi)* (*n* = 6) were compared to embryos from uninjected hermaphrodites (*n* = 70).
(1.2 MB PDF)

Figure S6 *pax-1* Is Expressed Independently of *htz-1*
(A) Onset of *pax-1::GFP* expression after the two-cell stage*. pax-1::GFP* expression was not influenced by microinjection of *htz-1* dsRNA (*p* = 0.8622, Student *t*-test. No RNAi: *n* = 23 embryos; *htz-1* RNAi: *n* = 12 affected embryos).
(B) Percentage of embryos containing one or more co-localized LacI::CFP and YFP::HTZ-1 dots in the pharynx (black) or outside of the pharynx (grey). No significant difference in pharyngeal HTZ-1 association was found when comparing the no-target lines (Figure 4) to the *pax-1* line (*p* > 0.1704, Fisher exact test).A total of 60 embryos were scored for the *pax-1* line. Images were taken at the bean, 1.5-, 2-, and 3-fold stages of embryogenesis. *pax-1* is activated at the comma stage by GFP reporter (J. Stevenson, A. Chisholm, S. E. Mango, unpublished data).
(211 KB PDF)

### Accession Numbers

The National Center for Biotechnology Information (NCBI) (http://www.ncbi.nlm.nih.gov) accession numbers for the genes discussed in the paper are as follows: *ama-1* (GeneID 177190), *hsf-1* (GeneID 173078), *htz-1(RNAi)* (GeneID 177212), *lin-26* (GeneID 3565051), *myo-2* (GeneID 181404), *mys-1* (GeneID 179096), *pax-1* (GeneID 187104), *pha-4* (GeneID 180357), *R07B1.9* (GeneID 181201), *smg-1(cc546ts)* (GeneID 172418), *ssl-1* (GeneID 190954), *tbx-2* (GeneID 175698), and *unc-54,* (GeneID 259839).

## Abbreviations

dsRNA: double-stranded RNA
L1: first larval stage
