# Supplementary figures and images for: Temporal Regulation of Foregut Development by HTZ-1/H2A.Z and PHA-4/FoxA
Source: PLoS Genet. 2006 Sep 29;2(9):e161. doi: 10.1371/journal.pgen.0020161 (PMC1584275; doi:10.1371/journal.pgen.0020161)

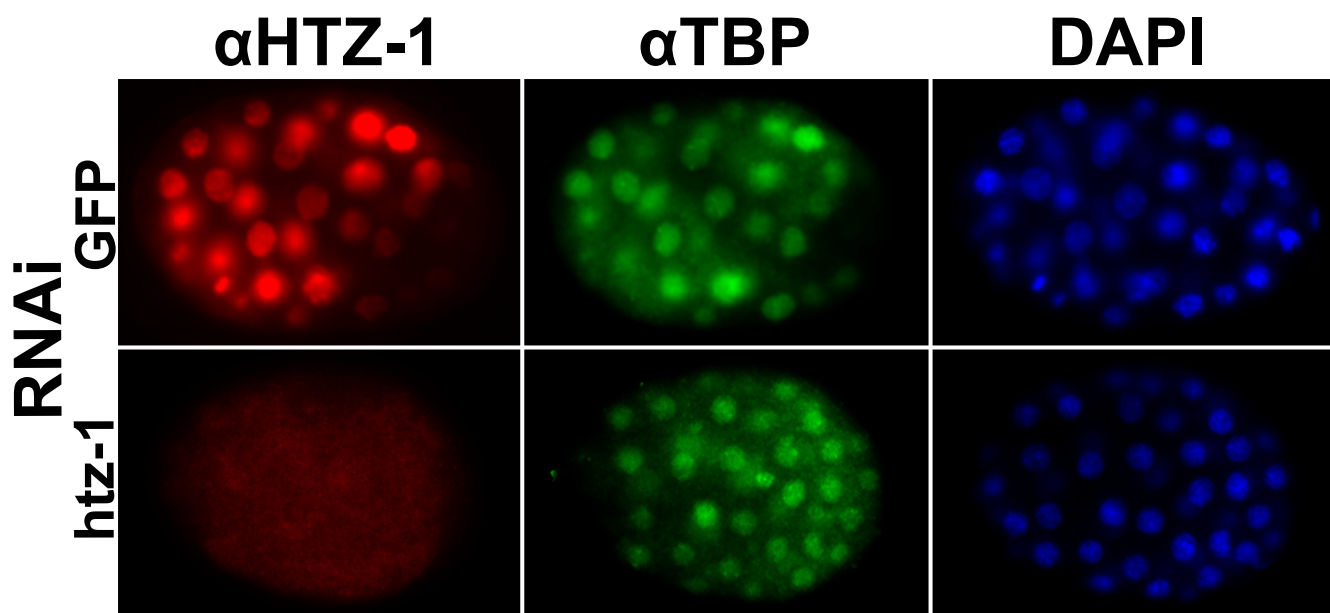

Supplement: Figure S1 — Cells co-stained with rabbit anti-TBP [65] and DAPI (n = 5). HTZ-1 antibody was a generous donation from Bill Kelly, Emory University; B. Kelly, unpublished data. (2.4 MB PDF) [file pgen.0020161.sg001.pdf]

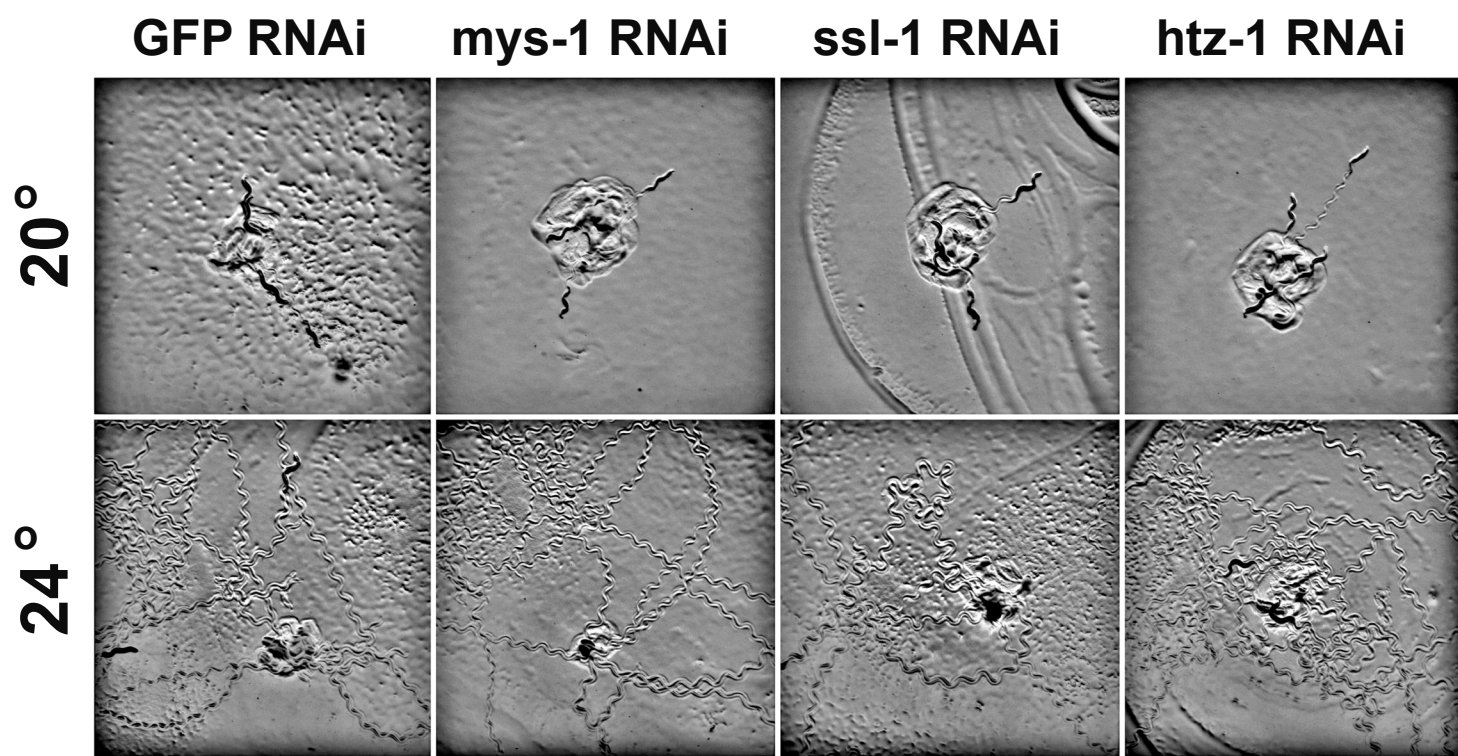

Supplement: Figure S2 — unc-54 activity was monitored by a crawling assay at the intermediate temperature of 20 °C or the permissive temperature of 24 °C. After RNAi against the indicated gene, four unc-54(ts) worms were picked to a new plate and allowed to crawl for 10 min. At 24 °C, unc-54(ts) worms were mobile, and this movement was not inhibited when mys-1, ssl-1, or htz-1 were inactivated. At 20 °C, unc-54(ts) worms were largely immobile, and movement was not improved by mys-1, ssl-1, or htz-1 RNAi. (4.7 MB PDF) [file pgen.0020161.sg002.pdf]

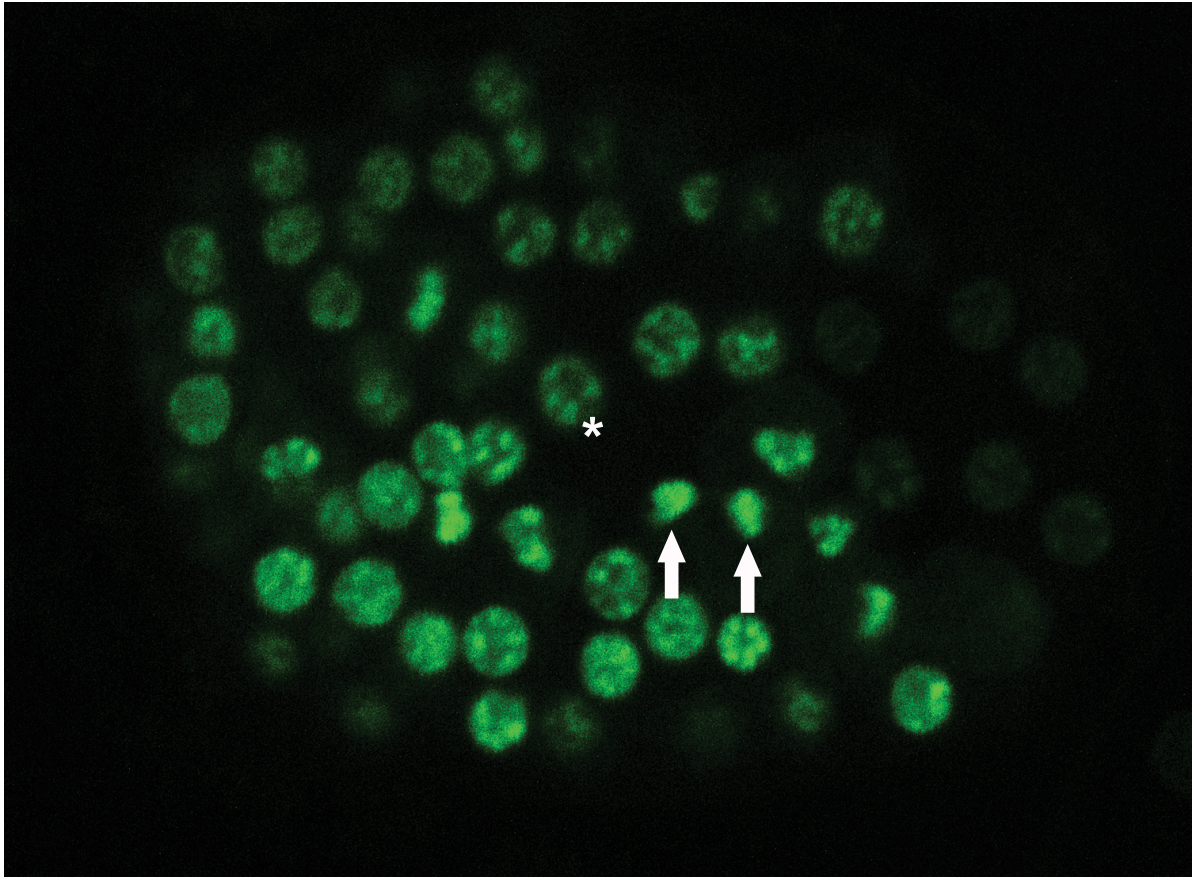

Supplement: Figure S3 — YFP::HTZ-1 associates with chromatin in cells during metaphase and anaphase (arrows). YFP::HTZ-1 is non-randomly distributed in cells during interphase (asterisk), similar to other organisms [10]. (2.9 MB PDF) [file pgen.0020161.sg003.pdf]

**A**

**htz-1 RNAi**

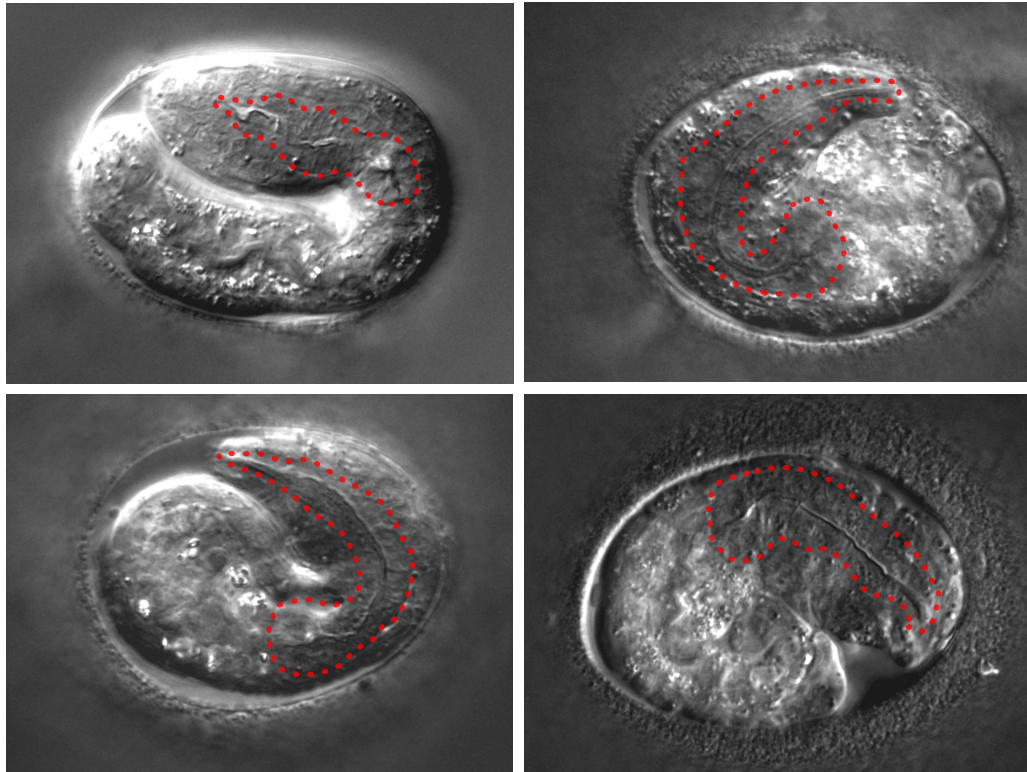

**B**

**ssl-1 RNAi**

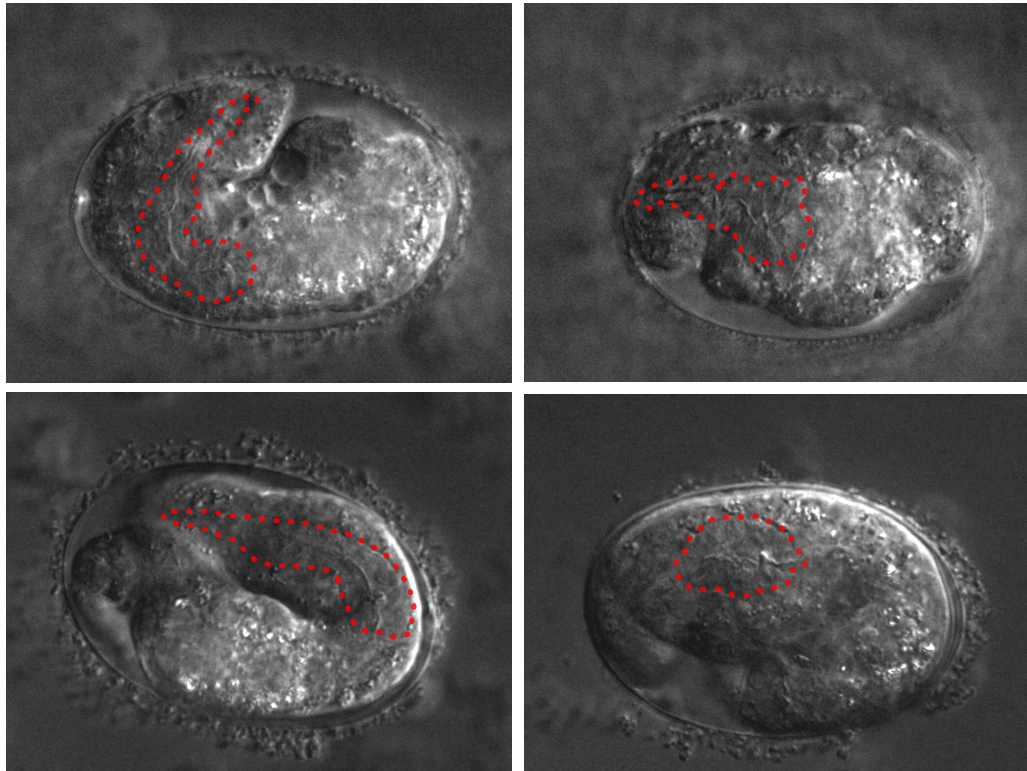

Supplement: Figure S4 — (A) Microinjection of 3.2 μg/μl of htz-1 dsRNA caused a highly penetrant late-embryonic arrest. Four terminal htz-1(RNAi) embryos show the pharynx (dotted red line) and intestine appear differentiated, but misshapen morphologically. (B) Microinjection of 0.5 μg/μl of ssl-1 dsRNA caused a highly penetrant late-embryonic arrest that is indistinguishable from htz-1(RNAi) under the light microscope. Four terminal ssl-1(RNAi) embryos show that the pharynx (dotted-red line) and intestine appear differentiated, but misshapen morphologically. Embryos from injected worms were picked to a new plate after 1 d and allowed to develop 18 h before imaging. (8.3 MB PDF) [file pgen.0020161.sg004.pdf]

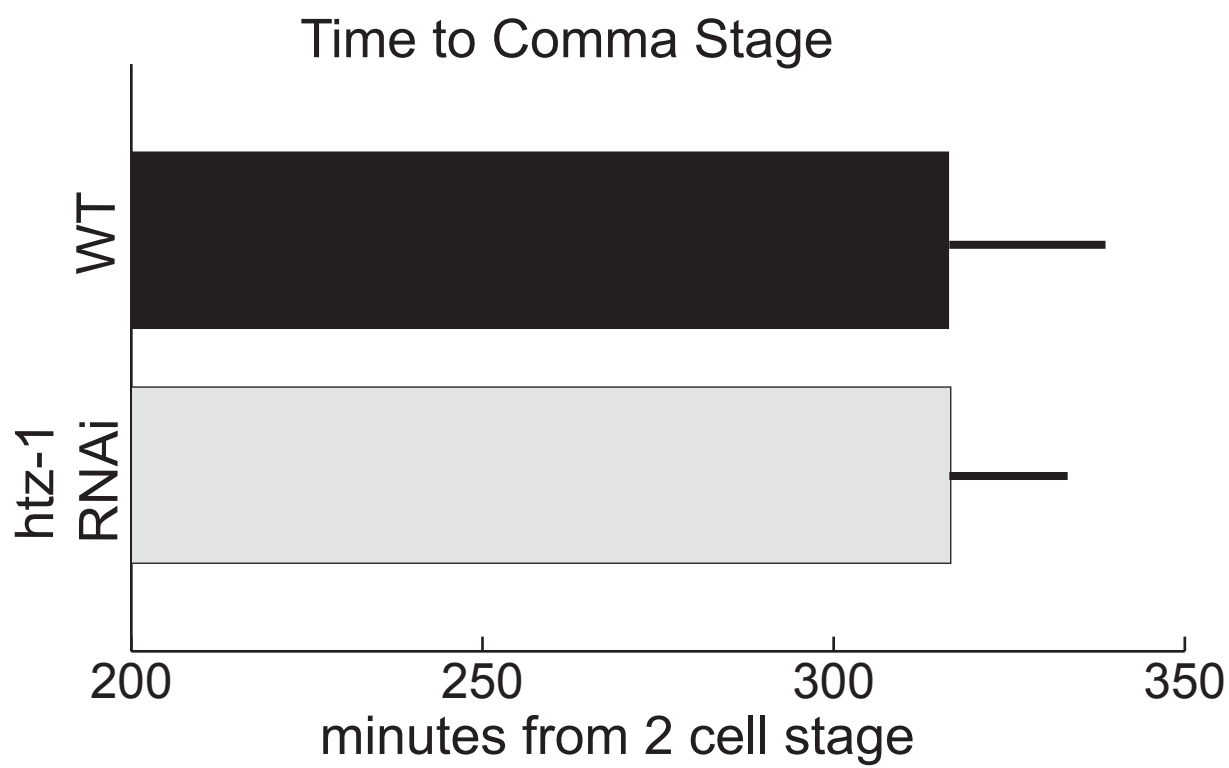

Supplement: Figure S5 — Two-cell embryos were dissected from uninjected hermaphrodites and hermaphrodites injected 2 d prior with 3.2 μg/μl of double-stranded htz-1 RNA. Only embryos with embryonic defects from htz-1(RNAi) (n = 6) were compared to embryos from uninjected hermaphrodites (n = 70). (1.2 MB PDF) [file pgen.0020161.sg005.pdf]

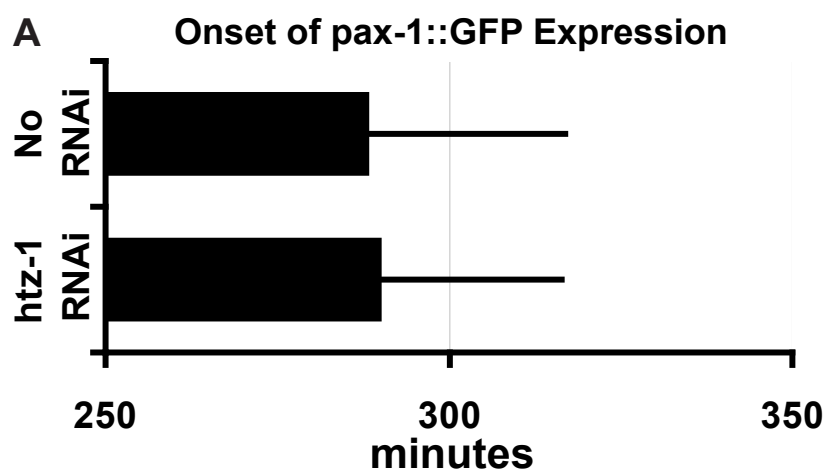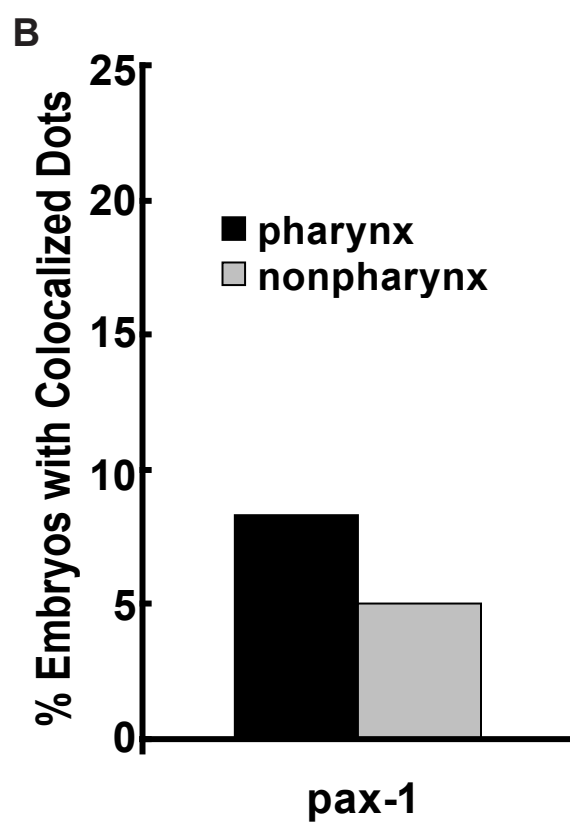

Supplement: Figure S6 — (A) Onset of pax-1::GFP expression after the two-cell stage. pax-1::GFP expression was not influenced by microinjection of htz-1 dsRNA (p = 0.8622, Student t-test. No RNAi: n = 23 embryos; htz-1 RNAi: n = 12 affected embryos). (B) Percentage of embryos containing one or more co-localized LacI::CFP and YFP::HTZ-1 dots in the pharynx (black) or outside of the pharynx (grey). No significant difference in pharyngeal HTZ-1 association was found when comparing the no-target lines (Figure 4) to the pax-1 line (p > 0.1704, Fisher exact test).A total of 60 embryos were scored for the pax-1 line. Images were taken at the bean, 1.5-, 2-, and 3-fold stages of embryogenesis. pax-1 is activated at the comma stage by GFP reporter (J. Stevenson, A. Chisholm, S. E. Mango, unpublished data). (211 KB PDF) [file pgen.0020161.sg006.pdf]
